# Biophysical and Solution Structure Analysis of Critical Residues Involved in the Interaction between the PupB N-Terminal Signaling Domain and PupR C-Terminal Cell Surface Signaling Domain from *Pseudomonas capeferrum*

**DOI:** 10.3390/biom14091108

**Published:** 2024-09-03

**Authors:** Tajnin Sultana, David M. Morgan, Beau D. Jernberg, Peyton Zak, Sangita C. Sinha, Christopher L. Colbert

**Affiliations:** 1 Department of Chemistry and Biochemistry, North Dakota State University, Fargo, ND 58108, USA; tajnin.sultana@ndsu.edu (T.S.); dmmorgan@gmail.com (D.M.M.); bjernberg18@gmail.com (B.D.J.); peytonzak6@gmail.com (P.Z.); 2 Independent Researcher, Winnipeg, MB R3C 0Z5, Canada; 3 Bio-Techne Corporation, 614 McKinley Place NE, Minneapolis, MN 55413, USA

**Keywords:** N-terminal signaling domain (NTSD), C-terminal CSS domain (CCSSD), NMR, ITC, CD, HSQC

## Abstract

**Abstract:** Cell surface signaling (CSS) is a means of rapidly adjusting transcription in response to extracellular stimuli in Gram-negative bacteria. The pseudobactin BN7/8 uptake (Pup) system not only imports iron but also upregulates its own transcription through CSS in *Pseudomonas capeferrum*. In the absence of ferric pseudobactin BN7/8, the signaling components are maintained in a resting state via the formation of a periplasmic complex between the N-terminal signaling domain (NTSD) of the outer membrane iron-transporter, PupB, and the C-terminal CSS domain (CCSSD) of the sigma regulator, PupR. The previously determined 1.6 Å crystal structure of this periplasmic complex has allowed us to probe the structural and thermodynamic consequences of mutating key interfacial residues. In this report, we describe the solution structure of the PupB NTSD and use Nuclear Magnetic Resonance spectroscopy, Isothermal Titration Calorimetry, and Circular Dichroism spectroscopy together with thermal denaturation to investigate whether three PupB point mutations, Q69K, H72D, and L74A, influence the interaction merely due to the chemical nature of the amino acid substitution or also cause changes in overall protein structure. Our results demonstrate that binding to the PupR CCSSD does not alter the structure of PupB NTSD and that the individual mutations have only minor effects on structure. The mutations generally lower thermodynamic stability of the NTSD and weaken binding to the CCSSD. These findings validate the X-ray crystal structure interface, emphasizing the importance of amino acid chemical nature at the interface.

## 1. Introduction

Iron is necessary for all living cells. However, the extremely low solubility of iron makes acquisition from the environment difficult. To overcome this, Gram-negative bacteria secrete iron-chelating molecules, called siderophores, to actively scavenge iron from their surroundings and then import them through a conserved process involving TonB-dependent transporters [1]. A subset of these TonB-dependent transporters controls their own transcription through a process known as cell surface signaling (CSS). CSS results in a rapid transcriptional response to environmental stimuli. Key components of iron import CSS pathways are (1) an outer membrane TonB-dependent transporter/transducer, which transports metabolites and transduces the external signal to the periplasm; (2) an inner membrane sigma regulator, also known as an inner membrane anti-sigma factor, which conveys the signal from the periplasm to the cytoplasm; and (3) a cytoplasmic sigma factor, which, when released, recruits RNA polymerase to initiate transcription of the response gene(s) [2]. Besides iron import, CSS systems are associated with biofilm formation, intercellular interactions, and release of virulence factors, in addition to metabolite transfer and regulation [3].

The best characterized CSS iron import systems are the *Pseudomonas capeferrum* (formerly *Pseudomonas putida* WCS358) ferric pseudobactin BN7/8 uptake system (Pup) [4,5], the *Escherichia coli* ferric citrate (Fec) transport system [6,7,8], and the *Pseudomonas aeruginosa* ferric pyoverdine (Fpv) import system [3,9,10]. Each of these homologous systems contains a TonB-dependent transporter/transducer that has an N-terminal signaling domain (NTSD) that precedes the transporter plug and barrel domains. Indeed, several NTSDs have been structurally characterized by either NMR spectroscopy, FecA and PupA, or X-ray crystallography, FpvA and PupB [9,11,12,13,14,15,16]. Each NTSD structure has the characteristic conserved βαβ–βαββ fold. The FecA NTSD has been shown to be responsible for conveying the transporter occupancy to the sigma-regulator, FecR [8,17]. Further, the complex between the PupB NTSD and periplasmic C-terminal cell surface signaling domain (CCSSD) of its cognate sigma-regulator, PupR, has also recently been structurally and biochemically characterized [16].

Interestingly, the homologous *P. capeferrum* TonB-dependent transporter, PupA, that shares 36% sequence identity with PupB, is signaling incompetent [4,5]. The PupA NTSD is unable to convey a signal to the periplasm. Further, a chimeric transporter/transducer, wherein the PupA NTSD was replaced by the PupB NTSD, was active for CSS in response to the binding of the ferric siderophore, pseudobactin 358 [5]. The characterization of the PupB NTSD:PupR CCSSD complex revealed that the presence of the NTSD stabilized the CCSSD and these two domains interact with low micromolar binding affinity, (K_d_ = 0.69 [0.42, 1.11] mM), wherein values in square brackets indicate a 68.3% asymmetric profile likelihood confidence interval for the mean value presented [16]. The structure of the PupB NTSD:PupR CCSSD complex enabled identification of PupB NTSD residues at the interface. A comparison of these PupB NTSD interface residues with residues in equivalent positions in the PupA NTSD should provide insights as to how destabilizing this interaction can influence CSS activation. Two interface PupB residues, H72 and Q69, were mutated to D and K respectively, the residues found at equivalent positions in the PupA NTSD. Additionally, as the PupA equivalent of the PupB interface residue L74 is also an L, L74 was mutated to A to significantly decrease the hydrophobic interaction at this site. Qualitatively, it was shown that the PupB NTSD H72D and L74A mutations disrupted the interaction with the PupR CCSSD, but the PupB NTSD Q69K mutation did not significantly alter the interaction [16]. However, these mutants were not characterized further. Thus, it is not known if the structure of the PupB NTSD H72D and L74A mutants is significantly altered relative to that of the wild-type (WT) PupB NTSD and the Q69K mutant.

Here, we report the Nuclear Magnetic Resonance (NMR) solution structure of the PupB NTSD and compare this structure to those of other known NTSDs, and also to the PupB NTSD structure found in the X-ray crystal structure of the complex with the PupR CCSSD. We use Isothermal Titration Calorimetry (ITC) to quantify and compare the thermodynamics of binding of the PupB NTSD Q79K, H72D, and L74A mutants to the PupR CCSSD to better evaluate how alteration of this interface influences the thermodynamics of interaction. Further, we use CD spectroscopy to compare the secondary structure content of the mutants to the WT NTSD and to evaluate how these mutations affect the thermal stability of the complex with the PupR CCSSD. Finally, we analyze the ^1^H/^15^N HSQC spectra of each of the three PupB NTSD mutants (H72D, L74A, and Q69K) to determine the extent to which these mutations alter structure relative to WT. Thus, this study provides insights into whether binding-associated conformational changes or the chemical nature of the interacting residues drive the interaction between the PupB NTSD and the PupR CCSSD during iron import CSS.

## 2. Materials and Methods

### 2.1. Cloning of the GST-PupB NTSD and MBP-PupR CCSSD and Mutagenesis

Expression plasmids encoding the PupB NTSD and the PupR CCSSD as N-terminally tagged glutathione S-transferase (GST-PupB NTSD) and maltose binding protein (MBP-PupR CCSSD) fusion proteins, respectively, that were previously described were used for this research [16,18]. Additionally, the GST-PupB NTSD region was excised from the pGEX vector and transferred to pET41b to create the pET41 GST-PupB NTSD plasmid, which enabled selection using kanamycin. Thus, both MBP-PupR CCSSD and GST-PupB NTSD can be co-expressed in the same bacteria. Each plasmid encodes a TEV protease recognition site between the N-terminal tag and the PupB NTSD or PupR CCSSD, allowing each protein to be purified with or without the affinity tag. Individual point mutations of the PupB NTSD (H72D, Q69K, and L74A) were made by site-directed mutagenesis of the pET41 GST-PupB NTSD plasmid using a QuikChange II kit (Agilent, Santa Clara, CA, USA) as previously described [16,18].

### 2.2. Expression and Purification of PupB NTSD and Mutants

Proteins used for ITC and CD analysis (PupB NTSD, PupB NTSD Q69K, PupB NTSD H72D, PupB NTSD L74A, and PupR CCSSD) were expressed and purified as previously described [16]. However, for NMR spectroscopy, *E. coli* BL21(DE3) pLysS cells were transformed with the pGEX-PupB NTSD WT vector. Positive transformants were selected by plating on LB (Luria Broth) agar with 100 μg/mL ampicillin. Expression of the GST-PupB NTSD fusion protein was carried out by the addition of 70 mL of overnight culture grown in LB plus ampicillin to prewarmed M9 minimal medium containing 3 g/L [U–^13^C_6_]-d-glucose and 1 g/L ^15^NH_4_Cl (Cambridge Isotope Laboratories, Inc., Tewksbury, MA, USA) with 100 μg/mL ampicillin and purified as previously described [16].

Mutant proteins for use in NMR were expressed in *E. coli* BL21(DE3) pLysS cells transformed with pET41b-PupB NTSD mutant vectors. Transformed cells were plated on LB agar plates with 25 μg/mL kanamycin. Overnight cultures were added to M9 minimal medium containing 1 g/L ^15^NH_4_Cl (Cambridge Isotope Laboratories, Inc., Tewksbury, MA, USA) with 25 μg/mL kanamycin. Cultures were grown at 37 °C with shaking at 200 rpm until they reached an OD_600_ = 0.6–0.8. Then, overnight expression at 20 °C was induced with 0.5 mM IPTG. Cells were harvested by centrifugation at 4000× *g* and cell pellets stored at −80 °C until use. U–^15^N labelled PupB NTSD or NTSD mutant cell pellets were resuspended in lysis buffer (25 mM Tris pH 7.8, 150 mM NaCl, 2 mM DTT) and disrupted by sonication (Branson Sonifier 450) followed by centrifugation at 20,000× *g*. Clarified lysate was loaded on a glutathione agarose gravity column prepacked with 10 mL of resin. The column was washed with 10 column volumes of lysis buffer at a flow rate of 0.5 mL/min. Tobacco Etch Virus protease was added to the column in an estimated 1:10 molar ratio for on-column cleavage of the GST-tag [19]. Free PupB NTSD was then washed from the column with the addition of 10 column volumes of lysis buffer and analyzed via SDS-PAGE. The PupB NTSD was further purified by gel filtration on a Superdex 75 10/300 column (Cytiva, Marlborough, MA, USA) equilibrated with lysis buffer without DTT. Finally, the samples were concentrated using a 3 K centrifugal concentrator (Millipore, Jaffrey, NH, USA) to 10–15 mg/mL (1.0–1.5 mM) before use.

### 2.3. Quantification of the MBP-PupR CCSSD Interaction with PupB NTSD Mutants

ITC was performed using a Low Volume Nano ITC (TA Instruments, New Castle, DE, USA). Purified proteins were loaded into separate dialysis cassettes (Thermo Scientific, Norristown, PA, USA) and co-dialyzed against 25 mM HEPES pH 7.5, 400 mM LiCl, 10% glycerol. Experiments were performed at 15 °C by titrating MBP-PupR CCSSD at concentration ranging between 140–230 µM into individual PupB NTSD mutants at concentrations ranging between 20–70 µM. MBP-PupR CCSSD was used for its favorable solubility and stability characteristics, as reported for our previous ITC experiments with the WT proteins [16]. Titrations comprised either 20 2.5 µL injections or 25 2 µL injections. All experiments were performed in triplicate. The values from a buffer-into-buffer titration were subtracted from the values of the protein-into-protein titration during analysis. Data were initially analyzed using NanoAnalyze (TA Instruments) using an independent, single-site model before export and analysis using NITPIC [20] for data integration, followed by processing with SEDPHAT [21] and plotting of isotherms in GUSSI 2.1.0 [22,23].

### 2.4. CD Spectroscopy and Thermal Denaturation of PupB NTSD Mutants

PupB NTSD Q69K, H72D, and L74A were each dialyzed separately against a solution of consisting of 10 mM potassium phosphate pH 6.8, 100 mM (NH_4_)_2_SO_4_ overnight at 4 °C and diluted to 25 µM. Continuous scanning CD spectra were measured at 4 °C from 190–250 nm using a Jasco J-710 spectrometer with a PTC-423S Peltier cell holder and a 1 mm quartz cell. Spectra were buffer subtracted and secondary structure content estimated using CONTIN and CDSSTR within the CDPro software package [24]. CD melting and re-folding curves from 25 µM of each PupB NTSD mutant were recorded at 217 nm between 10–85 °C in 1 °C increments with a slope of 1 °C/min, during both heating and cooling. Heating and cooling thermal denaturation data for each protein were fit to a 6-parameter version of the van’t Hoff relationship modified to include sloping baselines in the fully natured and fully denatured states:

$$\theta \left[T\right]=\frac{\left(b1+m1T\right)+\left(b2+m2T\right)e^{-\frac{\Delta H}{R}\left(\frac{1-T}{T_{m}}\right)}}{{1+e}^{-\frac{\Delta H}{R}\left(\frac{1-T}{T_{m}}\right)}}$$

in which *b*1 and *m*1 are the intercept and slope of the curve in the fully natured region, *b*2 and *m*2 are the intercept and slope in the fully denatured region, ΔH is the enthalpy of the denaturation reaction, *R* is the ideal gas constant, *T_m_* is the melting temperature (Kelvin), and *T* (Kelvin) is the independent variable. *T_m_* for each protein was determined by fitting this equation to the thermal denaturation data using a commercial version of Wolfram Mathematica (licensed to DMM). 

### 2.5. NMR Spectroscopy

#### 2.5.1. NMR Sample Preparation, Data Collection, and Chemical Shift Assignment

The suite of experiments used to obtain chemical shift assignments for WT PupB NTSD have been described previously, and the chemical shift assignments deposited [18]. Briefly, the samples consisted of 500 μL of 250–500 μM U–^15^N-labeled PupB NTSD, or 500 μM–1 mM U–^15^N/^13^C-labeled PupB NTSD, dialyzed against 50 mM Na phosphate pH 6.0, 100 mM NaCl overnight at 4 °C. D_2_O was added to a final concentration of 10% *v*/*v*. NMR data were recorded at 25 °C on Agilent DD2 spectrometers equipped with triple-resonance cryogenic probes and operating at 600 and 800 MHz. Backbone chemical shift assignments were initially performed semi-automatically using RunAbout in NMRViewJ [25,26], then transferred for continued manual assignment to CCPNmr [27]. Sequential assignment of backbone chemical shifts was accomplished with through-bond experiments: 3D HNCO, HNHA, HNCACB, and CBCA(CO)NH [28,29]. Side chain resonances were assigned from CBCA(CO)NH, H(CCO)NH, C(CO)NH, HCCH TOCSY, HBCB(CGCD)HD, and HBCB(CGCDCE)HE spectra [30]. Aromatic side chain assignments were obtained from HBCB(CGCD)HD, HBCB(CGCDCE)HE, and 2D ^1^H–^1^H NOESY experiments. To compile distance restraints, ^1^H–^15^N HSQC-NOESY were carried out at 600 MHz, and aromatic- and aliphatic-region ^1^H–^13^C HSQC-NOESY were carried out at 800 MHz. NOESY data were processed using standard nmrPipe scripts [31] and assembled into the CCPN data model, operating in the NMRBox environments [32]. Interproton distance constraints were obtained from 3D ^15^N edited NOESY (τ_m_ = 100 ms with 704 × 512 × 64 complex points), ^15^N, ^13^C edited NOESY (τ_m_ = 140 ms with 4096 × 156 × 256 complex points), and ^15^N edited 2D ^1^H–^1^H NOESY (τ_m_ = 100 ms with 2048 × 1024 complex points) spectra.

#### 2.5.2. NMR Hydrogen/Deuterium Exchange Experiments

300 μL of 834 μM uniformly ^15^N-labelled PupB NTSD in 100 mM sodium phosphate, pH 5.85, 100 mM NaCl was placed in a microcentrifuge tube and briefly spun. The amount of sodium phosphate was increased to maintain a buffering solution. At the original 50 mM phosphate concentration, the residual buffering capacity at pH 6.0 would be within an order of magnitude of the protein concentration for this experiment. Therefore, we doubled the concentration of sodium phosphate to 100 mM. Upon redissolution in 70% D_2_O, the sample was expected to have a pD = 6.0 [33], to match the pH used for the NMR experiments previously used for structure determination [18]. The microcentrifuge tube lid was punctured, the sample was frozen in liquid nitrogen, and subjected to lyophilization for 24 h. The tube was sealed with parafilm and kept at −80 °C until data collection at the Minnesota NMR Centre. There, the sample was redissolved in 300 mL of 70% D_2_O and briefly microcentrifuged. A 250 μL aliquot was withdrawn and used for data collection. ^1^H–^15^N NHSQC consisting of 2048 × 64 complex points, were implemented with 8 transients and a T_1_ relaxation delay of 1.7 s; each experiment lasted ~16 min. The first 13 experiments were collected sequentially, one immediately following the next, and a further 10 experiments were collected at one-hour intervals thereafter. The 0 s time point was defined as the midpoint date and time of the first experiment. Times elapsed between experiments thereafter were calculated as the difference of the midpoint time of the experiment in question and the midpoint time of the first experiment, for a total set of 23 time points spanning 0 through 65,684 s. These data were assembled into the CCPN data model for further analysis.

#### 2.5.3. Hydrogen Bond Restraints

Hydrogen/deuterium exchange experiments were processed using the “Data Analysis: Follow Intensity Changes” function in CCPN Analysis2.5. Intensities for each peak present in the zero-time spectrum were followed across the time series of ^1^H–^15^N HSQC spectra. Each intensity series was fit to a standard exponential decay function. To obtain hydrogen bond restraints, oxygen atom binding partners for each proton were inferred from the crystal structure of the PupB NTSD:PupR CCSSD complex. Putative proton-oxygen distances were set to a lower bound of 1.8 Å and an upper bound of 2.30 Å. The hydrogen bond restraints are listed in Table S1.

#### 2.5.4. Dihedral Angle Restraints

The CCPN implementation of DANGLE [34] was used to estimate dihedral angles; a maximum of two islands were allowed in Ramachandran space. These estimates were converted into restraints using the built-in function for doing so. The list of restraints is presented in the Supplementary Material, Table S2.

#### 2.5.5. NOE Assignment and Structure Calculation

ARIA2 [35] was used to carry out structure prediction and NOE assignment. Initial ARIA2 runs were constrained by the protein chemical shift list, the peak list from the ^15^N-edited proton NOE experiment with ~100 amide resonances assigned, and DANGLE restraints. The results of these calculations were subjected to manual violation analysis to further improve NOE assignments. Iterative rounds of ARIA2 calculation and violation analysis were carried out until ~90% of the ^15^N-edited NOE peaks were assigned. Hereafter, a template structure was added to all ARIA2 jobs. That structure consisted of the protein’s coordinates from the crystal structure of the PupB NTSD:PupR CCSSD complex (PDBID: 6OVK). Riding hydrogens as well as missing N- and C-terminal atoms were added using Chimera-X [36]. Hereafter, hydrogen bond restraints were also included as input to ARIA2 jobs. Then, iterative rounds of ARIA2 calculations and manual violation analysis were carried out with progressively larger ^13^C-filtered proton NOE peak lists until a suitably large and internally consistent dataset emerged to define the structure.

#### 2.5.6. Structure Validation

Structural validation was carried out with Procheck (1996) [37] and WhatIF [38] and the results of these analyses are presented in Table 1 and Table S3, respectively.

#### 2.5.7. ^1^H–^15^N HSQC Spectroscopy of Labelled PupB NTSD Mutants

^1^H–^15^N-HSQC experiments were recorded on samples at concentrations of 100–350 μM on a Bruker AMX spectrometer operating at a proton frequency of 400 MHz, with 2048 × 256 complex points in each dimension. The extent of signal averaging was varied according to the concentrations of the individual samples. Spectra were processed using standard NMRPipe scripts and incorporated into the CCPN data model using Analysis2.5 for further study. Peak shifts upon mutation were calculated by the formula [39]:

$$∆\partial =\sqrt{∆\partial H^{2}+0.1∆\partial N^{2}}$$

The peak shift for each mutant is listed in Table S4.

## 3. Results

### 3.1. Solution NMR Structure of the PupB NTSD

**Figure 1:**
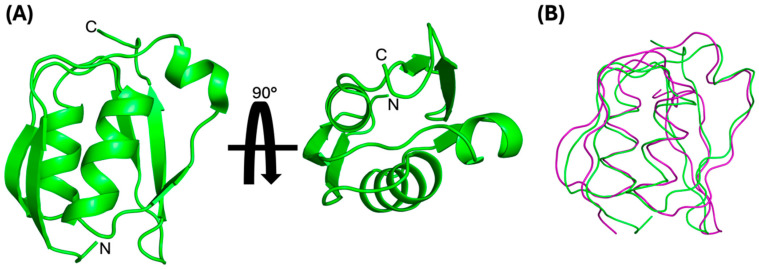
(**A**) Two orthogonal views of the average NMR solution structure of the PupB NTSD showing the conserved βαβ-βαββ fold. (**B**) NMR solution structure of PupB NTSD superimposed on the PupB NTSD from the crystal structure of the PupB NTSD:PupR CCSSD complex (PDBID: 6OVM). This and all molecular figures were made with PyMOL [42].

We determined the solution structure of the PupB NTSD using standard double- and triple-resonance NMR experiments conducted on uniformly ^15^N and ^15^N/^13^C labeled protein samples (Figure 1). This structure is based on 1662 geometric constraints obtained from measurements of interproton distances, dihedral angle estimates, and hydrogen exchange data (Table 1). These data are well satisfied by the high-precision ensemble of the 10 lowest-energy structures (Figure S1). With respect to their positions in the average NMR structure (Figure 1A), the set of protons restrained by the 1493 unambiguous and ambiguous distance restraints have RMSD = 0.500 ± 0.037 Å. The 10 lowest energy structures have been deposited with the RCSB/PDB database (accession no. 9CUV).

The solution structure exhibits the characteristic conserved βαβ-βαββ structure typical for Secretin TonB short N-terminal domains (STN domains, SMART accession number: SM00965) [40,41]. Comparison, using the PDB pairwise alignment tool (Available online: https://www.rcsb.org/alignment (accessed on 30 May 2024)), of our average solution structure to the solution structures of NTSDs from *E. coli* FecA (PDBID: 2D1U, [11]) and *P. capeferrum* PupA (PDBID: 2A02, [12]) as well as to the X-ray crystal structure of the NTSD from *P. aeruginosa* FpvA (PDBID: 2O5P, [14]) shows that our average solution structure is in strong agreement (rms deviation values range from 2.19–2.45 Å) with representative NTSDs (Figure S2). Further, the PupB NTSD average solution structure superimposed on the X-ray crystal structure of the PupB NTSD:PupR CCSSD complex (PDBID: 6OVK [16]) with an rms deviation of 1.4 Å over 71 out of 82 C_α_s, indicating no significant structural deviations, with only minor deviations in the loop regions. Thus, the structure of the PupB NTSD is minimally altered when in complex with the PupR CCSSD, relative to its unbound solution structure (Figure 1B).

### 3.2. Effect of Mutations on Interaction between the NTSD and the CCSSD

**Figure 2:**
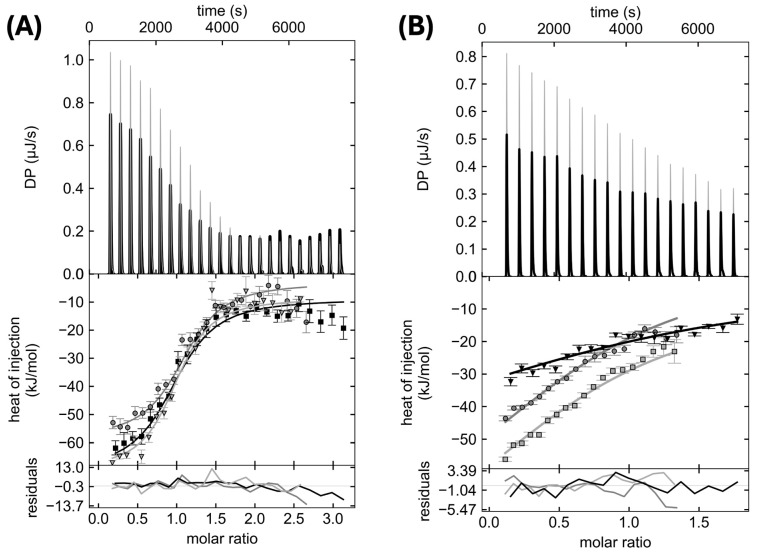
Global analysis of ITC isotherms for (**A**) PupB NTSD Q69K or (**B**) PupB NTSD H72D titrated into MBP-PupR CCSSD. The heats of binding (top panel), the isotherms with the curves for the global model (middle panel), and residuals of the global model fit (bottom panel) for the triplicate experiments are shown in black, gray, and light gray.

Previously, the WT PupB NTSD and MBP-PupR CCSSD were shown to bind with a dissociation constant (K_d_) of 0.69 μM [0.42, 1.11 μM] using ITC [16]. This interaction is driven by enthalpy rather than entropy. MBP-PupR CCSSD was used for its favorable solubility and stability characteristics, and MBP was verified to have no detectable interaction with the free PupR CCSSD [16]. Therefore, we used ITC to assess the impact of mutating three PupB interface residues on binding to the MBP-PupR CCSSD (Figure 2). Two of the residues selected for mutagenesis, Q69 and H72, were mutated to K and D respectively, which are the equivalent residues in the homologous signaling-incompetent PupA NTSD [16]. A third residue, L74, which is also an L in the PupA NTSD, was mutated to Ala.

In the crystal structure of the complex, the NTSD Q69 ε-amino protons hydrogen bond across the binding interface with the A240 carbonyl oxygen and the Q249 ε-oxygen in the CCSSD. We hypothesized that the Q69K mutation would disrupt these interactions and further insert a positive charge into a space otherwise occupied by the main chain atoms of A244, much of A246, and the γ-methyl group of V253 in the CCSSD. Surprisingly, our ITC results indicate this mutation has limited effect, causing only approximately two-fold decrease in binding affinity to a K_d_ of 1.55 [0.9, 2.6] µM (Table 2, Figure 2A). Notably, however, this mutation does impose an enthalpic penalty on binding, which is compensated by a nearly equivalent entropic gain. 

In contrast, the H72D mutation decreases the affinity between the NTSD and the CCSSD by greater than 39-fold to a K_d_ of 27.71 [U, 125] µM (Table 2, Figure 2B). This mutation provides a significant enthalpic gain and a large entropic penalty. NTSD residue H72 is involved in a salt-bridge with CCSSD residue E292, and also packs against CCSSD residues Q249 and M251. This suggests that the salt-bridge present in the WT interaction is a critical element for molecular recognition and high-affinity binding between the two protein domains.

The L74A mutation completely abrogates binding between the protein domains. Both ITC (Table 2) and the previously reported pull-down assay [16] did not detect binding. In the complex, NTSD residue L74 makes extensive hydrophobic contacts with CCSSD residues T288, F289, P290, A300, and the aliphatic part of the R296 CCSSD. This indicates that these hydrophobic interactions are critical for binding. 

### 3.3. Effect of NTSD Mutations on Folding and Thermal Stability

We used CD spectroscopy to assess and compare the overall secondary structure content of each mutant PupB NTSD to WT PupB NTSD (Table 3, Figure S3). Overall, the CD spectra of each mutant matched that for the WT PupB NTSD. The spectra have characteristic negative values between 205 nm and approximately 250 nm indicative of alpha helical and beta strand secondary structure. The lack of double negative peaks at 210 nm and 222 nm indicates the presence of significant beta structure in addition to helical content (Figure S3). Overall, the 82-residue WT NTSD was estimated to have 16 ± 1 residues in helical conformations, 30 ± 1 residue in strand conformations, and 36 ± 0 residues in coil conformations (Table 3), which unexpectedly was markedly different than that calculated from the average NMR solution structure above, which has 27, 22 and 33 residues in helical, strand and coil conformations, respectively. Notably, the secondary structure content of the WT NTSD NMR structure is similar to that observed in the NTSD from the crystal structure of the PupB NTSD:PupR CCSSD complex, consistent with overall low rmsds between the two structures. The Q69K mutant had 10 ± 3, 31 ± 4, and 41 ± 1 residues in helical, strand and coil, respectively (Table 3). H72D had 9 ± 3, 31 ± 2, and 42 ± 1 residues in helical, strand and coil (Table 3), while L74A had 17 ± 1, 30 ± 1, and 35 ± 1 residues in alpha, beta, and coil (Table 3). Relative to the WT NTSD, the secondary structure content of the L74A mutant is very similar, while the H72D and Q69K mutants appear to have marginally reduced helicity and correspondingly increased coil content. However, it is unclear if this difference is significant, given the error inherent in secondary structure estimations from CD, and the large difference between secondary structure content estimated by CD and the experimental NMR and X-ray crystal structures. Therefore, subsequently, as described in the next section, we analyzed the structures of the mutants using NMR.

We also used CD to monitor and compare the thermal stability of each mutant PupB NTSD to the WT (Table 3 and Table S5, Figure S4). The WT PupB NTSD has a T_m_ = 57.4 °C (heating) and a T_m_ = 53.8 °C (cooling). In contrast to the WT PupB NTSD, the PupB NTSD mutants have similar T_m_s in both the heating and cooling. All T_m_s are within 5 °C of each other (Table 3 and Table S5, Figure S4). H72D has the lowest T_m_ at 53 °C (Table 3), which, while not a large change, may represent a minimal disruption to the overall structure and decrease in stability. However, in general, the thermal stability data indicate that the overall stability of the PupB NTSD is undisturbed by the amino acid substitutions. Combined with the secondary structure analyses, this suggests that the overall PupB NTSD structure is unaffected by the Q69K, H72D, and L74A mutations. 

### 3.4. Comparison of ^1^H–^15^N HSQC Mutant and WT Spectra Indicates the Structural Effects of Mutation Are Modest

**Figure 3:**
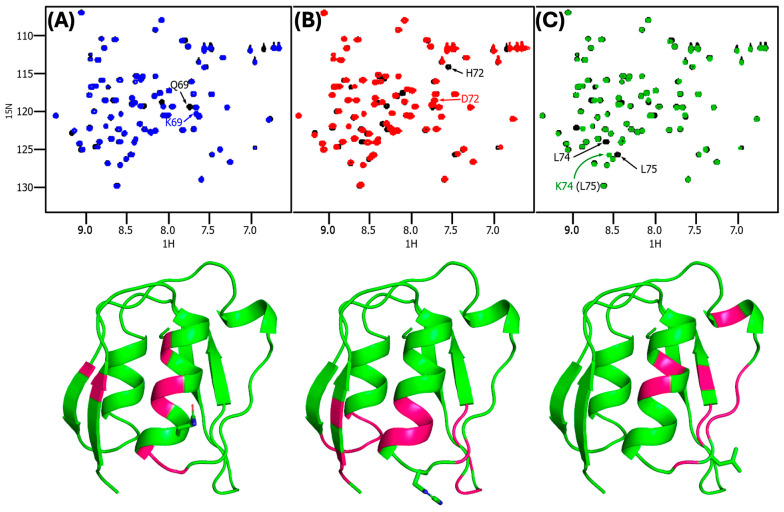
Superimposed ^1^H/^15^N HSQC spectra showing significant chemical shift changes between the WT PupB NTSD and the PupB NTSD mutants (top panels) with residues whose Δδ chemical shift change is ≥0.05 ppm highlighted for WT PupB NTSD (black contours) and (**A**) PupB NTSD Q69K (blue contours), (**B**) PupB NTSD H72D (red contours) and (**C**) PupB NTSD L74A (green contours). The position of each residue is indicated in each bottom panel by representation of the appropriate side chain.

We measured the ^1^H–^15^N HSQC spectra of the Q79K, H72D, and L74A NTSD mutants and compared them to the spectrum of the WT NTSD to identify any detailed changes in structure and to confirm that mutating interface residues did not impact overall structure (Figure 3). The PupB NTSD proteins used for these NMR experiments have 82 residues of which three are Pro, which are not detected in a ^1^H–^15^N HSQC spectrum. HSQC spectra for each of the three PupB NTSD mutants overlap substantially with that of the WT, indicating the three mutant proteins retain a very similar structure to the WT. Below, we discuss the peak shifts for each mutant individually.

The spectrum of the Q69K mutant is the most similar to WT, containing only two peaks which differ in position by Δδ ≥ 0.05 ppm, representing 2.5% of the backbone amide signal. Relative to the WT PupB NTSD spectrum, the spectrum of the Q69K mutant does not include either the Q69 backbone amide signal at 7.71 ^1^H ppm, 119.3 ^15^N ppm, or the signals from the ε-amido nitrogen atom and its attached protons. Instead, a signal not present in the WT spectrum is found at 7.68 ^1^H ppm, 120.3 ^15^N ppm, which we identify as the mutant lysine backbone signal (Figure 3A). Further, the mutant signal for H66 has ∆δ = 0.2 with respect to the WT, and the F67 signal has ∆δ = 0.1; other peaks shifted by smaller amounts. These residues are in the direct vicinity of the mutation (Figure 3A—bottom), suggesting that the structural effect of this mutation is modest and local, and distal to the binding interface.

The spectrum of the PupB NTSD L74A mutant contains five peaks which differ in position by ∆δ ≥ 0.05 ppm, representing 8.8% of the backbone amide signal. Relative to the WT PupB NTSD spectrum, in the spectrum of the L74A mutant, the L74 backbone amide signal at 8.59 ^1^H ppm, 123.9 ^15^N ppm is absent, while a signal absent in the WT spectrum is found at 8.57 ^1^H ppm, 125.7 ^15^N ppm, which we tentatively identify as the mutant alanine backbone amide resonances (Figure 3C). However, it is also possible that this signal is due to L75, which has shifted to a new position in the mutant, as in the WT spectrum it is located nearby, at 8.47 ^1^H ppm, 125.7 ^15^N ppm. Peaks which have changed due to the L74A mutation are: L121 (∆δ = 0.380), H66 (∆δ = 0.079), Y77 (∆δ = 0.063), I73 (∆δ = 0.060), and perhaps L75, although ∆δ was not calculated for L75 because of the aforementioned uncertainty in the identity of the putative L75 peak. These residues map to β-strands β3 and β4, whose residues have extensive hydrophobic contacts with each other (Figure 3C—bottom). This suggests subtle readjustment of the core packing in response to the mutation.

The peak shift is most pronounced in the spectrum of the H72D mutant, with 12 (15%) of backbone amide peaks changing by at least 0.05 ppm. Relative to the WT PupB NTSD spectrum, the spectrum of the H72D mutant lacks the backbone amide resonance for H72 at 7.56 ^1^H ppm, ^15^N 114.1 ppm, and a new signal is present at 8.25 ^1^H ppm, ^15^N 120.2 ppm, which we attribute to the mutant aspartic acid backbone amide resonances (Figure 3B). Peak shifts are much more widespread in this mutant spectrum than in the other two mutants discussed and include the following peaks: S48 (Dd = 0.159), Q50 (∆δ = 0.154), A51 (∆δ = 0.136), F53 (∆δ = 0.085), F67 (∆δ = 0.077), G68 (∆δ = 0.070, Q69 (∆δ = 0.07), S70 (∆δ = 0.059), L74 (∆δ = 0.056), D95 (∆δ = 0.055), I96 (∆δ = 0.053), and D97 (∆δ = 0.051). This implies that the chemical environments of these backbone amides have all been influenced by the H72D mutation. Mapping these positions onto the structure (Figure 3C—bottom), reveals that these residues cluster across secondary structural elements adjacent to the vicinity of the mutation. This suggests that the overall structure of the NTSD is similar, and the chemical shifts of these residues are altered due to interactions with the mutated residue. The NMR evidence suggests that, on the μs–ms timescale of NMR, the mutant occupies a closely related, but slightly altered structure. As discussed above, the H72D mutant has a lower thermal stability, which is consistent with a slightly greater disruption of structure.

Thus, our comparison of the WT and mutant PupB NTSD ^1^H–^15^N HSQC spectra allows us to conclude with confidence that, although the mutations cause modest local perturbation in the structure, the overall structure is largely preserved in the case of all three mutants. The Q69K and L74A mutants show negligible perturbation of structure; therefore, the two-fold decrease in binding affinity in the former and complete abrogation of binding in the latter can clearly be attributed to the changes in the chemical nature of residues upon mutation, rather than due to structural changes that may have taken place upon mutation. We are less certain of this with respect to the H72D mutant, but the totality of the chemical shift changes is within standard acceptable chemical shift ranges for each residue with respect to structural changes (Available online: https://bmrb.io/ref_info/csstats.php (accessed on 30 June 2024)). Thus, we conclude it is the chemical nature of the amino acid side chain in the NTSD that is critical for driving the association with the CCSSD.

## 4. Discussion

The solution NMR structure of the PupB NTSD reported here demonstrates that the NTSD structure is unaffected by its interaction with the PupR CCSSD. Our previously reported analysis of the interaction between the PupB NTSD and the PupR CCSSD identified PupB NTSD residues Q69, H72, and L74 as being involved in the interface [16]. Subsequent mutation of Q69 and H72 to the corresponding residues in the signaling incompetent PupA NTSD (Q69K and H72D), as well as an L74A mutation, were qualitatively tested for their influence on the PupB NTSD:PupR CCSSD interaction [16]. Affinity pulldown assays indicated that the Q69K had a minimal, if any, effect on the interaction, while H72D and L74A each provided significant disruption to the complex. This is consistent with our ITC results, which show that the PupB NTSD H72D mutation weakened binding to the PupR CCSSD by > 39-fold weakening and the NTSD L74A mutation completely abrogated the NTSD:CCSSD interaction, but the NTSD Q69K mutation had little effect on binding affinity. The large enthalpic gain observed for binding of the H72D mutant may be because a water-mediated interaction is more easily maintained with the smaller side chain in the NTSD H72D mutant, while the large entropic penalty may be because the H72D side chain carboxylate resides near the Sδ atom in the CCSSD M251 side chain. Together, this results in an overall decrease in the free energy of binding for this mutant. For the Q69K mutant, the enthalpic penalty is likely due to the loss of the interaction between the NTSD Q69 and the CCSSD Q249, which helps maintain the water-mediated interaction to CCSSD A240 carbonyl oxygen, with the entropic gain due to the aliphatic portion of the Lys side chain in the Q69K mutant packing better with CCSSD A246. Therefore, the free energy of binding remains similar. Thus, the mutations investigated likely disrupted complex formation due to the different chemical nature of the mutated residues preventing interaction rather than protein conformational changes.

Our CD analysis shows that neither the total secondary structure content nor the thermal stability of the PupB NTSD is significantly altered by the Q69K, H72D, and L74A mutations, indicating there is no large-scale change in the structures of the mutants. Our comparative analyses of the ^1^H–^15^N HSQC spectra of the WT PupB NTSD and the Q69K, H72D, and L74A PupB NTSD mutants verify that the overall folding of the mutant proteins is unaffected, although the mutants exhibit differing extents of local, atomic-level changes. The Q69K mutant showed the least structural perturbation, consistent with our results indicating that this mutation has little effect on binding. Part of NTSD residue Q69 is solvent exposed in the complex, so it is likely that the mutant lysine side chain is more easily accommodated in the complex. Among the three mutants, the H72D mutant involves the most drastic change in the chemical nature of the residue, i.e., from basic to acidic, and shows the largest structural changes, yet it surprisingly still binds to the PupR CCSSD, albeit with substantially weaker affinity. The L74A mutant shows less structural perturbation than the H72D mutant, and the chemical change due to this mutation is not as drastic, yet this mutation completely abrogates binding to the CCSSD, highlighting the importance of hydrophobic packing in stabilizing this interaction.

Thus, our results demonstrate that the structure of the PupB NTSD is not significantly altered upon binding to the CCSSD, indicating the NTSD binds with the CCSSD in a lock-and-key mechanism relative to the NTSD structure. Together our data also validate the interface delineated by the X-ray crystal structure of the PupB NTSD:PupR CCSSD complex. Importantly, our results unambiguously show that binding to the CCSSD is dramatically reduced or eliminated by mutating two NTSD residues at the interface, H72 to Asp, and L74 to a smaller Ala, due to the change in the chemical nature of these residues, even though these mutations do not significantly perturb overall structure. Future experiments involving hydrogen–deuterium exchange mass spectroscopy as well as surface plasmon resonance coupled to X-ray crystallographic results could further provide important insights into how the Q69K and H72D mutations alter the interaction interface or influence kinetic parameters, like k_on_ and k_off_, which could help further delineate the mechanism of interaction. Our results provide important atomic-level understanding of the mechanism of stabilization and priming of CSS in the ferric pseudobactin BN7/8 pathway in *P. capeferrum.* These results have broad implications in identifying the protein surfaces that dictate the stabilization of periplasmic complexes involved in other iron import CSS pathways.

## Figures and Tables

**Table 1:** Statistics for PupB NTSD solution structure determination.

List of Constraints	
NOE Distance Restraints:	
Unambiguous	1312
Ambiguous	181
Hydrogen Bond Restraints	50
Dihedral Angle Restraints	144
**Structural Analysis**	
Mean r.m.s.d. from experimental restraints:	
NOE (Å)	0.501 ± 0.031
Dihedral angles (deg.)	5.834 ± 0.927
Average number of:	
NOE violations > 0.5 Å	70.3 ± 2.4
NOE violations > 0.3 Å	99.9 ± 4.3
Dihedral violations > 5°	12.4 ± 1.7
Mean rms from idealized covalent geometry:	
Bonds, Å	0.0099 ± 0.0004
Angles, °	1.06 ± 0.03
Impropers, °	1.28 ± 0.06
Geometric analysis of residues 2–82	
Rmsd to mean, backbone, Å	0.79 ± 0.35
Rmsd to mean, all heavy, Å	1.46 ± 0.34
Ramachandran Analysis (Procheck)	
Most-favored region (%)	62.5
Additionally Allowed region (%)	33.4
Generously Allowed region (%)	3.9
Disallowed region (%)	0.15

**Table 2:** Thermodynamic parameters determined by ITC upon titrating MBP-PupR CCSSD into each PupB NTSD.

PupB NTSD	K_d_ (µM)	ΔH (kJ/mol)	ΔS (J/mol·K)	ΔG (kJ/mol)	Incompetent Fraction (%)
WT	0.69 [0.42, 1.11] ^a^	−73.99 [−80.99, −68.27] ^a^	−138.83 [−158.94, −122.93] ^a^	−33.99 [−35.18, −32.84] ^a^	0.0–12.8%
Q69K	1.55 [0.9, 2.6] ^a^	−60.70 [−67.83, −55.03] ^a^	−99.42 [−119.81, −84.05] ^a^	−32.05 [−33.31, −30.81] ^a^	0.0–9.3%
H72D	27.71 [U ^b^, 125.63] ^a^	−96.16 [U ^b^, −67.65] ^a^	−246.46 [U ^b^, −122.93] ^a^	−25.14 [U ^b^, −21.52] ^a^	0.0–43.5%
L74A	No Interaction Detected	-	-	-	-

^a^ Values in square brackets indicate a 68.3% c asymmetric profile likelihood confidence interval for the mean value presented. ^b^ U—Unbounded.

**Table 3:** Secondary structure (estimated percentage of residues) and melting temperature (T_m_) analysis of the WT PupB NTSD and its three mutants: Q69K, H72D, and L74A.

PupB NTSD	Helix	Strand	Coil + Turn	Total	Tm (Heating)	Tm (Cooling)
WT	19.5 ± 1.2	36.6 ± 1.2	43.9 ± 0	100	57.4 ± 0.1 °C	53.8 ± 0.9 °C
Q69K	12.2 ± 3.7	37.8 ± 4.9	50.0 ± 1.2	100	57.8 ± 0.9 °C	58.9 ± 0.1 °C
H72D	11.0 ± 3.7	37.8 ± 2.4	51.2 ± 1.2	100	53.1 ± 0.3 °C	53.3 ± 0.3 °C
L74A	20.7 ± 1.2	36.6 ± 1.2	42.7 ± 1.2	100	56.3 ± 0.2 °C	56.3 ± 0.2 °C

## Data Availability

The atomic coordinates for the ensemble of the 10 lowest energy structures of the PupB NTSD have been deposited into the Protein Data Bank (Available online: http://www.rcsb.org (accessed on 12 August 2024)) under PDB code 9CUV. The ^1^H, ^15^N, and ^13^C chemical shift assignments used for structure solution were previously deposited in the BioMagResBank (Available online: http://www.bmrb.wisc.edu (accessed on 8 November 2017)) under accession number 27141.s.

## Supplementary Materials

The following supporting information can be downloaded at: https://www.mdpi.com/article/10.3390/biom14091108/s1, Figure S1: Ensemble of the 10 lowest energy structures; Figure S2: Superposition of homologous NTSD structures on the PupB NTSD structure; Figure S3: CD spectra of WT PupB NTSD (black), PupB NTSD Q69K (purple), PupB NTSD H72D (green) and PupB NTSD L74A (red); Figure S4: Melting curves for PupB NTSD (A) WT; (B) Q69K; (C) H72D; and (D) L74A; Table S1: List of Hydrogen Bond Restraints; Table S2: List of Dihedral Angle Restraints; Table S3: Computation of Δδ between Peaks in Mutant and WT Spectra; Table S4: Summary of WhatIf Quality Parameters; Table S5: Best fit parameters for the 6-parameter version of the van’t Hoff relationship for each PupB NTSD thermal denaturation curve.

## List of Abbreviations

Cell surface signaling	CSS
C-terminal cell surface signaling domain	CCSSD
Circular dichroism spectroscopy	CD
Ferric citrate	Fec
Ferric pyoverdine	Fpv
Isothermal titration calorimetry	ITC
N-terminal signaling domain	NTSD
Nuclear magnetic resonance	NMR
Pseudobactin BN7/8 uptake	Pup
Wild-type	WT
