# Supplementary material for: Biophysical and Solution Structure Analysis of Critical Residues Involved in the Interaction between the PupB N-Terminal Signaling Domain and PupR C-Terminal Cell Surface Signaling Domain from Pseudomonas capeferrum
Source: Biomolecules. 2024 Sep 3;14(9):1108. doi: 10.3390/biom14091108 (PMC11429574; doi:10.3390/biom14091108)
Supplement: Supplementary file 1 [file biomolecules-14-01108-s001.zip › biomolecules-3112421-supplementary.pdf]

Supplementary Information

Biophysical and Solution Structure Analysis of Critical Residues Involved in the Interaction between the PupB N-terminal Signaling Domain and PupR C-terminal Cell Surface Signaling Domain from *Pseudomonas capeferrum.*

Authors: Tajnin Sultana, David M. Morgan, Beau D. Jernberg, Peyton Zak, Sangita S

inha, and Christopher L. Colbert

Supplemental Figures:

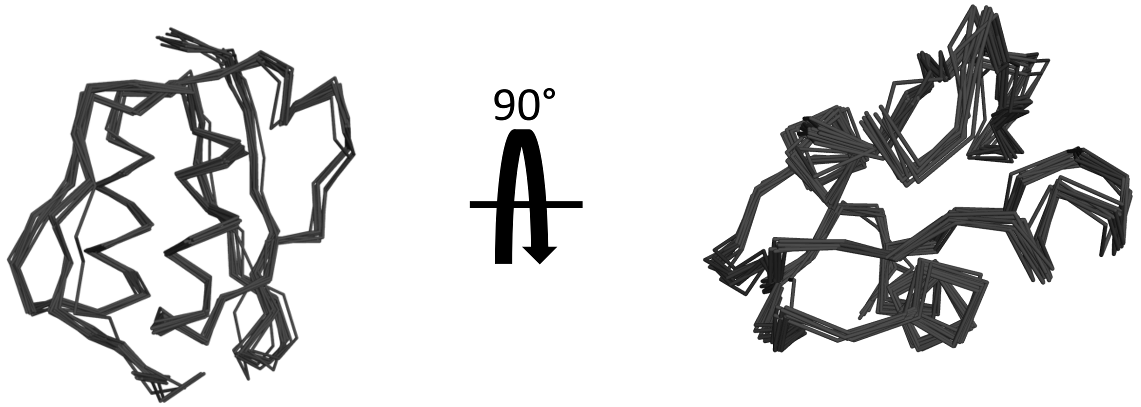

Figure S1. Ensemble of the 10 lowest energy structures. The ensemble is oriented as in Figure 1 with the same orthogonal view as in Figure 1(A). This ensemble has been deposited with the RCSB/PDB database (accession number 9CUV).

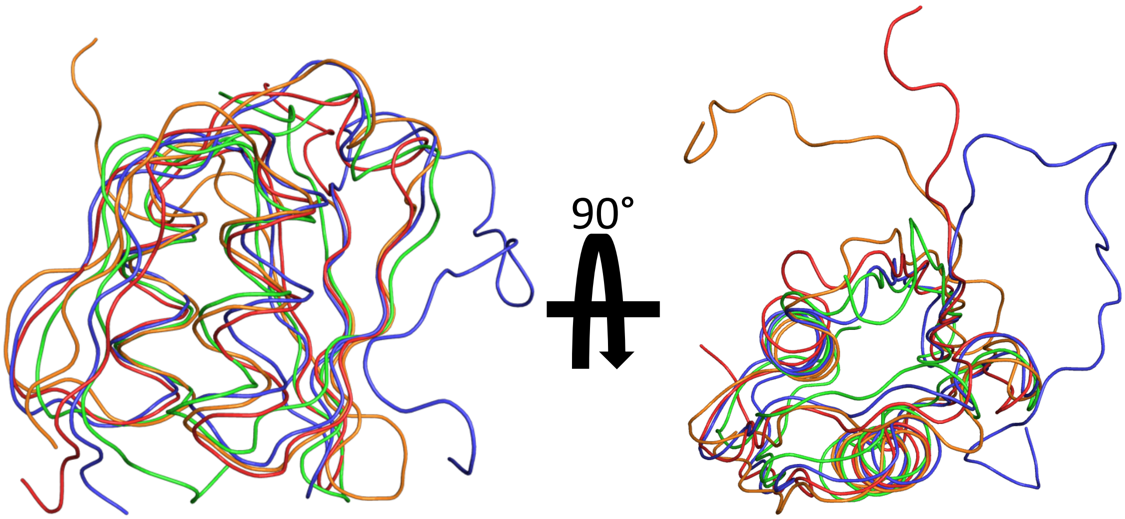

Figure S2. Superposition of homologous NTSD structures on the PupB NTSD structure. Average NMR solution structures from *P. capeferrum* PupA NTSD (PDBID: 2Ao2, red) and *E. coli* FecA NTSD NMR (PDBID: 2D1U, brown) as well as the structure of the NTSD from *P. aeruginosa* FpvA (PDBID: 2O5P, blue) were superimposed on the average PupB NTSD structure (green) using (https://www.rcsb.org/alignment). Views are as found in Figure 1.

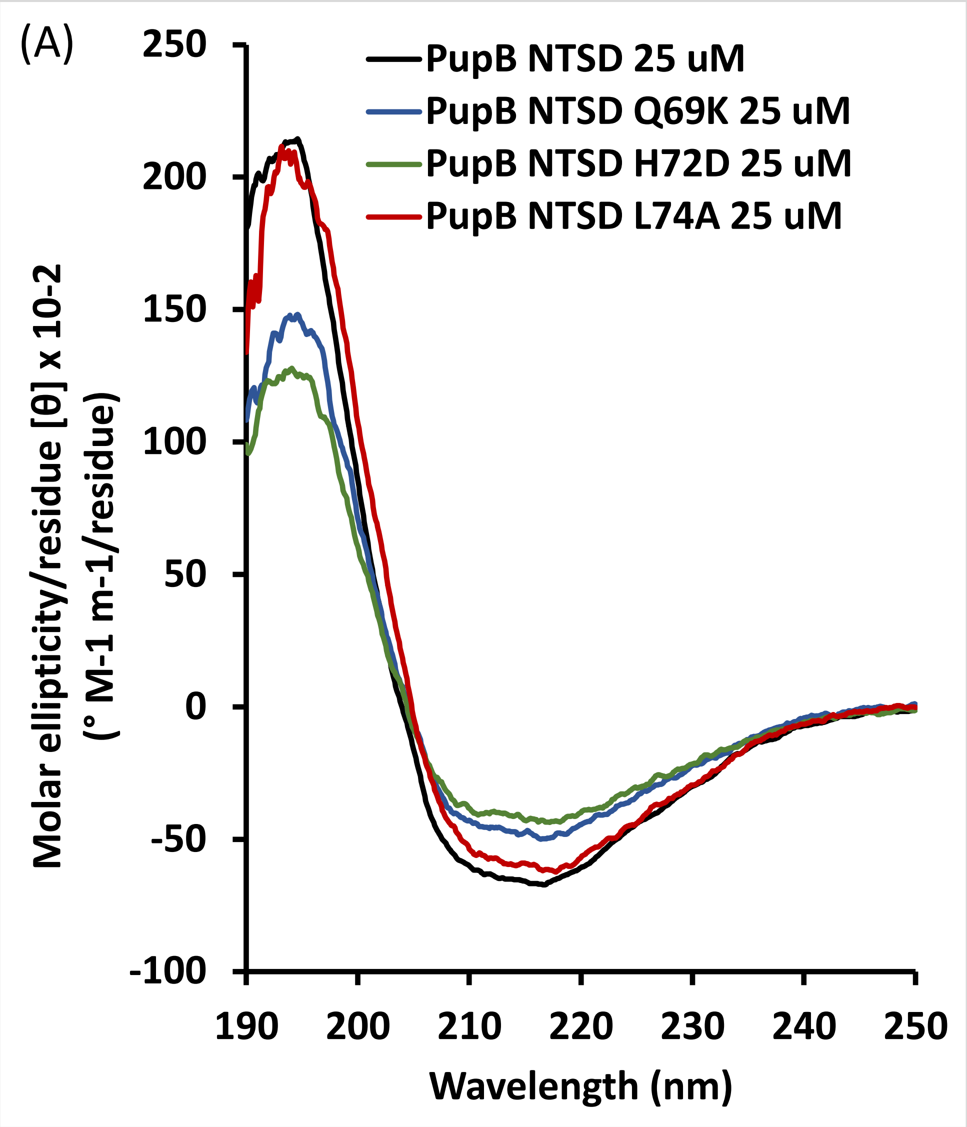

Figure S3. CD spectra of WT PupB NTSD (black), PupB NTSD Q69K (purple), PupB NTSD H72D (green) and PupB NTSD L74A (red).

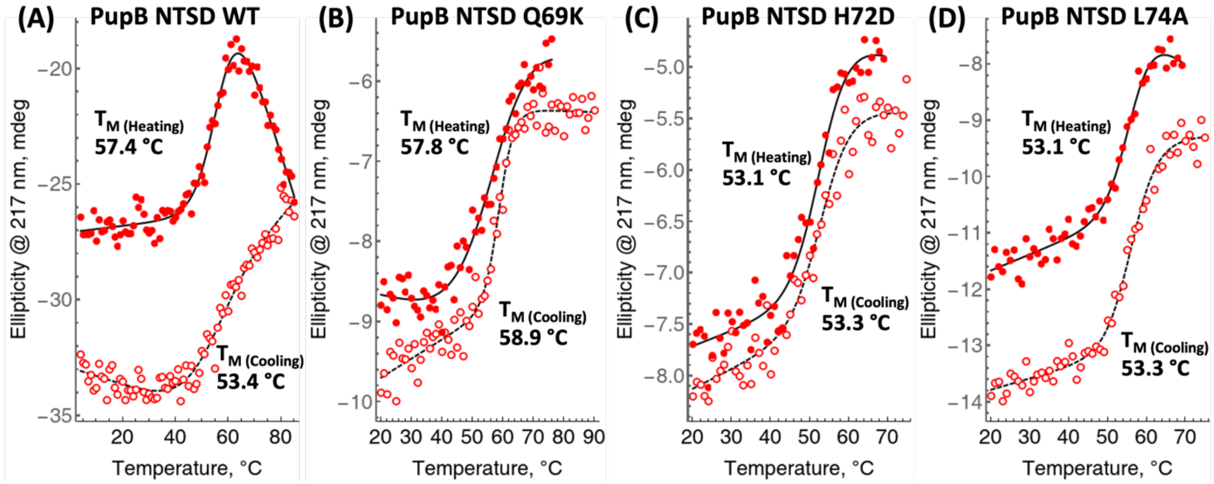

Figure S4. Melting curves for PupB NTSD (A) WT; (B) Q69K; (C) H72D; and (D) L74A. Unfolding (heating; red filled circles) and refolding (cooling; open circles) data points are shown. The fits to a 6-parameter version of the van't Hoff relationship are shown and T_ms are indicated.

Supplementary Tables:

Table S1: List of Hydrogen Bond Restraints

Residue A	Restrained Atom A	Residue B	Restrained Atom B	Distance, Å	Lower Error Bound, ± Å	Upper Error Bound, ± Å
7	HN	45	O	1.8	0	0.5
7	N	45	O	2.8	0	0.5
9	HN	44	O	1.8	0	0.5
9	N	44	O	2.8	0	0.5
12	HN	41	O	1.8	0	0.5
12	N	41	O	2.8	0	0.5
14	HN	39	O	1.8	0	0.5
14	N	39	O	2.8	0	0.5
17	HN	14	O	1.8	0	0.5
17	N	14	O	2.8	0	0.5
18	HN	14	O	1.8	0	0.5
18	N	14	O	2.8	0	0.5
19	HN	15	O	1.8	0	0.5
19	N	15	O	2.8	0	0.5
21	HN	17	O	1.8	0	0.5
21	N	17	O	2.8	0	0.5
25	HN	21	O	1.8	0	0.5
25	N	21	O	2.8	0	0.5
27	HN	22	O	1.8	0	0.5
27	N	22	O	2.8	0	0.5
41	HN	12	O	1.8	0	0.5
41	N	12	O	2.8	0	0.5
43	HN	41	OG	1.8	0	0.5
43	N	41	OG	2.8	0	0.5
44	HN	9	O	1.8	0	0.5
44	N	9	O	2.8	0	0.5
46	HN	7	O	1.8	0	0.5
46	N	7	O	2.8	0	0.5
48	HN	5	O	1.8	0	0.5
48	N	5	O	2.8	0	0.5
53	HN	49	O	1.8	0	0.5
53	N	49	O	2.8	0	0.5
54	HN	50	O	1.8	0	0.5

54	N	50	O	2.8	0	0.5
55	HN	51	O	1.8	0	0.5
55	N	51	O	2.8	0	0.5
56	HN	52	O	1.8	0	0.5
56	N	52	O	2.8	0	0.5
57	HN	53	O	1.8	0	0.5
57	N	53	O	2.8	0	0.5
58	HN	54	O	1.8	0	0.5
58	N	54	O	2.8	0	0.5
59	HN	56	O	1.8	0	0.5
59	N	56	O	2.8	0	0.5
63	HN	61	OG	1.8	0	0.5
63	N	61	OG	2.8	0	0.5
64	HN	76	O	1.8	0	0.5
64	N	76	O	2.8	0	0.5
76	HN	64	O	1.8	0	0.5
76	N	64	O	2.8	0	0.5

Table S2: List of Dihedral Angle Restraints

Restrained Atoms as 'ResidueNumber.AtomName'				Restraint Angle, °	Restraint Error Bound, ± °
2.C	3.N	3.CA	3.C	-86.65	53.35
3.N	3.CA	3.C	4.N	133.24	33.24
3.C	4.N	4.CA	4.C	-135.17	34.83
4.N	4.CA	4.C	5.N	155.08	35.08
4.C	5.N	5.CA	5.C	-135.49	34.51
5.N	5.CA	5.C	6.N	155.21	35.21
5.C	6.N	6.CA	6.C	-120.55	39.45
6.N	6.CA	6.C	7.N	137.01	37.01
6.C	7.N	7.CA	7.C	-124.86	35.14
7.N	7.CA	7.C	8.N	129.36	29.36
7.C	8.N	8.CA	8.C	-82.27	77.73
8.N	8.CA	8.C	9.N	130.73	40.73
8.C	9.N	9.CA	9.C	-106.89	53.11
9.N	9.CA	9.C	10.N	122.83	32.83
9.C	10.N	10.CA	10.C	-76.61	53.39
10.N	10.CA	10.C	11.N	161.34	41.34
10.C	11.N	11.CA	11.C	-67.77	62.23
11.N	11.CA	11.C	12.N	136.14	36.14
11.C	12.N	12.CA	12.C	-67.11	32.89
12.N	12.CA	12.C	13.N	138.21	38.21
12.C	13.N	13.CA	13.C	-64.83	25.17
13.N	13.CA	13.C	14.N	151.52	31.52
13.C	14.N	14.CA	14.C	-59.42	30.58
14.N	14.CA	14.C	15.N	-50.64	19.36
14.C	15.N	15.CA	15.C	-54.91	25.09
15.N	15.CA	15.C	16.N	-54.9	15.1
15.C	16.N	16.CA	16.C	-55.93	24.07
16.N	16.CA	16.C	17.N	-33.85	26.15
16.C	17.N	17.CA	17.C	-65.14	24.86
17.N	17.CA	17.C	18.N	-43.35	26.65
17.C	18.N	18.CA	18.C	-57.55	22.45
18.N	18.CA	18.C	19.N	-44.04	25.96
18.C	19.N	19.CA	19.C	-64.19	25.81
19.N	19.CA	19.C	20.N	-39.99	30.01
19.C	20.N	20.CA	20.C	-64.95	25.05
20.N	20.CA	20.C	21.N	-43.59	26.41
20.C	21.N	21.CA	21.C	-64.97	25.03
21.N	21.CA	21.C	22.N	-44.16	15.84

21.C	22.N	22.CA	22.C	-64.42	25.58
22.N	22.CA	22.C	23.N	-41.07	28.93
22.C	23.N	23.CA	23.C	-65.09	24.91
23.N	23.CA	23.C	24.N	-44.77	25.23
23.C	24.N	24.CA	24.C	-65.07	24.93
24.N	24.CA	24.C	25.N	-25.07	34.93
24.C	25.N	25.CA	25.C	-89.83	30.17
25.N	25.CA	25.C	26.N	-3.43	26.57
25.C	26.N	26.CA	26.C	55	15
26.N	26.CA	26.C	27.N	45	25
26.C	27.N	27.CA	27.C	-132.27	37.73
27.N	27.CA	27.C	28.N	155.18	35.18
27.C	28.N	28.CA	28.C	-113.94	36.06
28.N	28.CA	28.C	29.N	134.34	34.34
28.C	29.N	29.CA	29.C	-123.37	36.63
29.N	29.CA	29.C	30.N	136.82	36.82
29.C	30.N	30.CA	30.C	-124.43	35.57
30.N	30.CA	30.C	31.N	133.52	33.52
30.C	31.N	31.CA	31.C	-81.98	78.02
31.N	31.CA	31.C	32.N	150.79	60.79
31.C	32.N	32.CA	32.C	-63.73	16.27
32.N	32.CA	32.C	33.N	144.9	24.9
32.C	33.N	33.CA	33.C	-55.67	24.33
33.N	33.CA	33.C	34.N	-34.24	25.76
33.C	34.N	34.CA	34.C	-65.01	24.99
34.N	34.CA	34.C	35.N	-25.22	44.78
34.C	35.N	35.CA	35.C	-75.26	44.74
35.N	35.CA	35.C	36.N	-13.74	56.26
35.C	36.N	36.CA	36.C	-60.71	29.29
36.N	36.CA	36.C	37.N	-24.23	35.77
36.C	37.N	37.CA	37.C	-88.86	41.14
37.N	37.CA	37.C	38.N	4.41	44.41
38.C	39.N	39.CA	39.C	-116.04	43.96
39.N	39.CA	39.C	40.N	139.44	29.44
39.C	40.N	40.CA	40.C	-137	33
40.N	40.CA	40.C	41.N	154.68	44.68
40.C	41.N	41.CA	41.C	-116.19	43.81
41.N	41.CA	41.C	42.N	126.25	36.25
41.C	42.N	42.CA	42.C	-75.89	44.11
42.N	42.CA	42.C	43.N	125.09	25.09
42.C	43.N	43.CA	43.C	-143.82	26.18
43.N	43.CA	43.C	44.N	163.9	43.9
43.C	44.N	44.CA	44.C	-137.85	32.15

44.N	44.CA	44.C	45.N	142	32
44.C	45.N	45.CA	45.C	-126.11	33.89
45.N	45.CA	45.C	46.N	138.47	38.47
45.C	46.N	46.CA	46.C	-122.79	37.21
46.N	46.CA	46.C	47.N	138.28	28.28
46.C	47.N	47.CA	47.C	-119.91	40.09
47.N	47.CA	47.C	48.N	125.67	25.67
47.C	48.N	48.CA	48.C	-129.82	30.18
48.N	48.CA	48.C	49.N	158.94	38.94
49.C	50.N	50.CA	50.C	-56.97	33.03
50.N	50.CA	50.C	51.N	-42.56	27.44
50.C	51.N	51.CA	51.C	-64.79	25.21
51.N	51.CA	51.C	52.N	-42.16	27.84
51.C	52.N	52.CA	52.C	-64.94	25.06
52.N	52.CA	52.C	53.N	-43.61	16.39
52.C	53.N	53.CA	53.C	-63.91	26.09
53.N	53.CA	53.C	54.N	-42.98	27.02
53.C	54.N	54.CA	54.C	-62.32	17.68
54.N	54.CA	54.C	55.N	-42.19	27.81
54.C	55.N	55.CA	55.C	-64.71	25.29
55.N	55.CA	55.C	56.N	-38.82	21.18
55.C	56.N	56.CA	56.C	-65.16	24.84
56.N	56.CA	56.C	57.N	-44.47	15.53
56.C	57.N	57.CA	57.C	-64.98	25.02
57.N	57.CA	57.C	58.N	-25.4	34.6
57.C	58.N	58.CA	58.C	-94.5	25.5
58.N	58.CA	58.C	59.N	4.11	24.11
58.C	59.N	59.CA	59.C	-60.11	19.89
59.N	59.CA	59.C	60.N	134.84	24.84
59.C	60.N	60.CA	60.C	-71.49	38.51
60.N	60.CA	60.C	61.N	-15.02	44.98
60.C	61.N	61.CA	61.C	-69.28	30.72
61.N	61.CA	61.C	62.N	145.08	35.08
61.C	62.N	62.CA	62.C	75	25
62.N	62.CA	62.C	63.N	15	25
62.C	63.N	63.CA	63.C	-124.83	35.17
63.N	63.CA	63.C	64.N	139.43	39.43
63.C	64.N	64.CA	64.C	-135.04	34.96
64.N	64.CA	64.C	65.N	145.55	15.55
64.C	65.N	65.CA	65.C	-125.01	24.99
65.N	65.CA	65.C	66.N	134.92	24.92
65.C	66.N	66.CA	66.C	-121.23	38.77
66.N	66.CA	66.C	67.N	131.09	31.09

66.C	67.N	67.CA	67.C	-78.69	41.31
67.N	67.CA	67.C	68.N	129.43	39.43
68.C	69.N	69.CA	69.C	-73.5	46.5
69.N	69.CA	69.C	70.N	-15.39	34.61
70.C	71.N	71.CA	71.C	64.69	24.69
71.N	71.CA	71.C	72.N	25.6	25.6
71.C	72.N	72.CA	72.C	-116.08	53.92
72.N	72.CA	72.C	73.N	132.63	32.63
72.C	73.N	73.CA	73.C	-124.08	35.92
73.N	73.CA	73.C	74.N	139.79	29.79
73.C	74.N	74.CA	74.C	-126.45	43.55
74.N	74.CA	74.C	75.N	143.43	33.43
74.C	75.N	75.CA	75.C	-125.97	34.03
75.N	75.CA	75.C	76.N	150.85	40.85
75.C	76.N	76.CA	76.C	-135.53	44.47
76.N	76.CA	76.C	77.N	158.33	48.33
76.C	77.N	77.CA	77.C	-82.81	47.19
77.N	77.CA	77.C	78.N	128.65	38.65
77.C	78.N	78.CA	78.C	-60.18	39.82
78.N	78.CA	78.C	79.N	136.78	36.78

Table S3: Computation of $\Delta\delta$ between Peaks in Mutant and WT Spectra.

Mutant	Shifted Peak	$\Delta\delta$, ppm	$\Delta\delta \geq$ threshold?	H ppm, Mutant	N ppm, Mutant	H ppm, WT	N ppm, WT
Q69K	D54	0.031	No	8.883	124.67	8.904	124.6
	A63	0.035	No	6.794	121.08	6.778	120.98
	H66	0.200	Yes	8.076	119.38	8.096	118.75
	F67	0.100	Yes	9.162	122.5	9.193	122.8
	A71	0.040	No	8.357	119.28	8.317	119.27
	H72	0.028	No	7.582	114.18	7.562	114.12
	L90	0.026	No	8.683	118.54	8.657	118.54
H72D	S48	0.035	No	8.456	116.16	8.423	116.12
	Q50	0.159	Yes	8.065	117.08	8.121	117.55
	A51	0.056	Yes	8.273	122.19	8.307	122.33
	F53	0.024	No	8.899	119.94	8.919	119.98
	F67	0.085	Yes	9.194	123.11	9.192	122.84
	G68	0.154	Yes	8.848	105.7	8.873	105.22
	Q69	0.077	Yes	7.767	119.14	7.756	119.38
	S70	0.136	Yes	8.391	116.05	8.341	115.65
	L74	0.070	Yes	8.546	124.07	8.614	124.02
	D95	0.025	No	8.703	120.71	8.719	120.77
	I96	0.026	No	8.529	122.91	8.555	122.9
	D97	0.030	No	8.212	117.89	8.189	117.95
	Q98	0.051	Yes	8.319	121.55	8.361	121.64
	G114	0.055	Yes	8.421	115.14	8.41	115.31
	N116	0.053	Yes	8.704	116.7	8.752	116.77
	A117	0.059	Yes	8.371	118.76	8.428	118.81
	Y119	0.070	Yes	8.974	121.91	8.974	122.13
L74A	L64	0.032	No	8.462	119.1	8.463	119
	H66	0.079	Yes	8.099	118.51	8.096	118.76
	I73	0.060	Yes	7.835	110.8	7.816	110.62
	L75	0.101	Yes	8.574	125.73	8.474	125.68
	S76	0.033	No	8.756	118.55	8.788	118.52
	Y77	0.063	Yes	7.651	120.47	7.647	120.67
	L81	0.018	No	7.721	116.01	7.734	116.05
	Y119	0.035	No	8.894	122.25	8.8976	122.14
	L121	0.380	Yes	8.409	120.54	8.029	120.51

Table S4: Summary of WhatIf Quality Parameters

Quality Indices Generated by WhatIf

Checks Performed	Value	Error	Min	Max
1st generation packing quality Z-score (QUACHK)	-2.41	0.211	-2.77	-2.16
2nd generation packing quality Z-score (NQACHK)	-2.25	0.384	-2.87	-1.84
Ramachandran plot appearance Z-score (RAMCHK)	-7.42	0.478	-7.93	-6.25
Chi-1 chi-2 rotamer normality Z-score (C12CHK)	-7.6	0.203	-7.78	-7.1
Backbone conformation Z-score (BBCCHK)	-1.63	0.435	-2.19	-0.93
Bond lengths RMS Z-score (BNDCHK)	0.619	0.0291	0.578	0.672
Bond angles RMS Z-score (ANGCHK)	0.778	0.0254	0.73	0.812
Omega angle restraints RMS Z-score (OMECHK)	0.519	0.0244	0.461	0.551
Side chain planarity RMS Z-score (PLNCHK)	0.143	0.0465	0.078	0.234
Improper dihedral distribution RMS Z-score (HNDCHK)	0.788	0.0552	0.725	0.913
Inside/outside distribution RMS Z-score (INOCHK)	0.889	0.0094	0.875	0.904
Inter-atomic bumps (BMPCHK)	62.9	3.54	58	68
Unsatisfied hydrogen donors (BH2CHK)	10.9	3.03	6	15
Unsatisfied hydrogen acceptors (BA2CHK)	0.4	0.516	0	1

Table S5. Best fit parameters for the 6-parameter version of the van't Hoff relationship for each PupB NTSD thermal denaturation curve.

**NTSD →**	**WT**		**Q69K**		**H72D**		**L74A**	
**Param ↓**	**Heating**	**Cooling**	**Heating**	**Cooling**	**Heating**	**Cooling**	**Heating**	**Cooling**
**m1** (ε/K)	0.014 (54.4) [a]	-0.037 (41.2) [a]	-0.01 (156) [a]	0.025 (17.4) [a]	0.015 (61) [a]	0.019 (43.7) [a]	0.029 (22.6) [a]	0.02 (31.5) [a]
**b1** (ε)	-30.9 (7.2) [a]	-22.9 (19.1) [a]	-5.6 (86.4) [a]	-16.7 (7.6) [a]	-12 (22.5) [a]	-13.7 (9.8) [a]	-20 (9.8) [a]	-19.7 (9.8)
**m2** (ε/K)	-0.365 (7.1) [a]	0.096 (74.5) [a]	-0.028 (307) [a]	-0.001 (426) [a]	-0.016 (306) [a]	-0.003 (99.5) [a]	-0.063 (99.5) [a]	0.003 (1099) [a]
**b2** (ε)	105 (8.6) [a]	-60 (43.1) [a]	4 (706) [a]	-6 (38.5) [a]	1 (3025) [a]	-5 (168) [a]	14 (157) [a]	-10 (97.6)
**ΔH** (kJ/mol)	213 (19) [a]	112 (24.6) [a]	134 (30.9) [a]	371 (16.8) [a]	234 (28) [a]	230 (28) [a]	286 (20.1) [a]	268 (16.2) [a]
$\mathbf{T_m}$ (°C)	57.4 (0.2) [a]	53.8 (1.7) [a]	57.8 (1.7) [a]	58.9 (0.2) [a]	53.1 (0.5) [a]	53.3 (0.5) [a]	56.3 (0.4) [a]	56.3 (0.3) [a]
[a] Values in parenthesis are the percent (%) error for each parameter.								
